# REPERCUSSIONS OF BARIATRIC SURGERY ON METABOLIC PARAMETERS: EXPERIENCE OF 15-YEAR FOLLOW-UP IN A HOSPITAL IN MACEIÓ, BRAZIL

**DOI:** 10.1590/0102-672020210002e1627

**Published:** 2022-01-31

**Authors:** José Adailton PINHEIRO, Ingrid Ramalho Dantas de CASTRO, Ingrid Botelho RIBEIRO, Marcus Vinícius Quirino FERREIRA, Pérola Averbug FIREMAN, Marcos Antônio Duarte MADEIRO, Ana Carolina Pastl PONTES

**Affiliations:** 1 Hospital Memorial Arthur Ramos, Serviço de Cirurgia Bariátrica - Maceió - Alagoas - Brasil; 2 Centro Universitário Tiradentes-UNIT/AL, Curso de Medicina - Maceió - Alagoas - Brasil; 3 Centro Universitário CESMAC, Curso de Medicina - Maceió - Alagoas - Brasil

**Keywords:** Obesity, Bariatric Surgery, Metabolic Syndrome, Obesidade, Cirurgia Bariátrica, Síndrome Metabólica

## Abstract

Obesity is a disease characterized by an excessive accumulation of body fat, which is harmful to health, and it has grown significantly in the past years in the majority of countries. The surgery should be recommended to those patients with obesity who did not succeed in conservative clinical therapy after a rigorous analysis by a multidisciplinary team.

**Objective:**

The aim of this study was to compare metabolic results, weight loss, and parameters associated with obesity in the preoperative and postoperative periods of patients treated with bariatric surgery.

**Methods:**

This was a retrospective, descriptive, cross-sectional, and quantitative study through consultation medical records. Data were collected from May to September 2020 from individuals treated with bariatric surgery in a period of 15 years (from 2003 to 2018). A comparative and descriptive statistical analysis of anthropometric, metabolic, biochemical, and associated morbidities was performed.

**Results:**

The majority of patients were female (68.50%). In both sexes, the highest prevalence was found in the age group of 30-39 years and more than half had grade III obesity. The surgical technique used was gastroplasty with Roux-en-Y gastric bypass. After 4 months, there was a significant reduction in the lipid profile, anthropometric parameters, and liver enzymes in both sexes, which remained decreasing till the end of the first year, with marked improvement in the metabolic syndrome (MS).

**Conclusions:**

The positive impact resulted from gastroplasty in terms of weight loss, reduction of body mass index, and lipid profile is quite relevant after 4 months and it is maintained until 1 year after the procedure, showing benefits in reducing the risk factors of the MS.

## RESUMO - RACIONAL

A obesidade é caracterizada pelo acúmulo excessivo de gordura corporal prejudicial à saúde e nos últimos anos tem crescido significativamente na maioria dos países. A cirurgia bariátrica deverá ser recomendada para pacientes obesos que não obtiveram êxito no tratamento clínico e após análise da equipe multiprofissional.

**Objetivo:**

comparar os resultados metabólicos, perda ponderal e parâmetros associados à obesidade no pré e pós-operatório dos pacientes submetidos à cirurgia bariátrica.

**Métodos:**

estudo retrospectivo, descritivo, transversal, de abordagem quantitativa através da consulta a prontuários. Os dados foram coletados no período de maio a setembro de 2020, de indivíduos submetidos à cirurgia bariátrica no período de 15 anos (2003 a 2018). Foi realizada análise estatística comparativa e descritiva das variáveis antropométricas, metabólicas, bioquímicas e morbidades associadas.

**Resultados:**

a maioria era do sexo feminino (68,50%). Em ambos os sexos a maior prevalência se encontravam na faixa etária de 30 a 39 anos e mais da metade tinha obesidade grau III. A técnica cirúrgica utilizada foi o gastroplastia em Y de Roux. Após 4 meses houve uma redução significativa do perfil lipídico, dos parâmetros antropométricos e enzimas hepáticas em ambos os sexos, os quais permaneceram em declínio no final do primeiro ano, com melhora acentuada da síndrome metabólica.

**Conclusões:**

O impacto positivo determinado pela gastroplastia na perda de peso, na redução do IMC e perfil lipídico é bastante relevante já após quatro meses, e se mantém após um ano da realização do procedimento, demonstrando benefícios na redução dos fatores de risco da síndrome metabólica.

## INTRODUCTION

Obesity is a disease characterized by an excessive accumulation of body fat, which is harmful to health, and it has grown significantly in the past years in the majority of countries worldwide. This disease is responsible for a series of health complications that directly affect the quality of life and the life expectancy of individuals affected by it.^19^ The World Health Organization (WHO) defines its diagnosis as a body mass index (BMI) ≥30 kg/m^2^.

According to the Ministry of Health, Brazil, in 2016, noncommunicable diseases were responsible for 74% of the total deaths, which were caused mainly by cardiovascular diseases (28%), neoplasms (18%), respiratory diseases (6%), and diabetes (5%)^4^. During the period between 2013 and 2018, obesity, excessive body weight, and diabetes increased exceedingly.

Bariatric surgery is indicated for people with BMI >40 kg/m^2^ or BMI >35 kg/m^2^ who already presented certain pathologies caused or aggravated by obesity, according to the recommendations in the resolution Nº 2.131/2015 from the Federal Council of Medicine (CFM), in which 21 comorbidities are established. In addition to these indications, it is important to note the failure in clinical obesity therapy for at least 2 years^9^.

This requirement does not apply to individuals with BMI >50 kg/m^2^ ^9^. However, a new resolution of the CFM (Nº 2.172/2017) determined that patients with BMI between 30 and 34.9 kg/m^2^ and those who were diagnosed with refractory type 2 diabetes mellitus (DM) by two endocrinologists could undergo the surgery, which is then classified as a metabolic surgery in this situation, with Roux-en-Y gastric bypass (RYBG) being the first choice for the treatment^10^.

This intervention promotes dietary restriction through structural changes in the gastrointestinal tract. Its performance has been increasing currently and it has been considered an efficient method for the treatment of morbid obesity and for the long-term control of weight. In addition, it has also gained importance in the control of diabetes, reduction of risk factors for cardiovascular diseases and other comorbidities related to obesity, and the treatment of individuals with refractory metabolic syndrome (MS)^7^ ^,^ ^13^ ^,^ ^16^.

The surgery should be recommended to those patients with obesity who did not succeed in conservative clinical therapy (diet and medications) after a rigorous analysis by a multidisciplinary team, who will evaluate the nutritional status, psychological profile, and conditions. After the procedure, the patient must follow medical and nutritional recommendations, which should be complemented with a correct and individualized diet plan, nutritional supplementation, and physical activity. This is necessary because the success of the surgical therapy essentially depends on the emotional equilibrium and the changes in lifestyle and nutritional habits of an individual^8^ ^,^ ^21^.

Since the beginning of the therapy, the patients must be aware of their diagnosis of disease and be informed about the risks, benefits, and consequences of the surgery. Therefore, the aim of this study was to compare the metabolic results, weight loss, and parameters associated with obesity in the preoperative and postoperative periods of patients treated with bariatric surgery.

## METHODS

This was a retrospective, descriptive, cross-sectional, and quantitative study performed in an internal medicine clinic at the Hospital Memorial Arthur Ramos. This clinic conducts an average of eight bariatric surgeries monthly, which are performed by the same professional doctor and nutritionist, and is located in the city of Maceió, Alagoas.

Data were collected in the period between May and September 2020, using the medical records of patients treated with bariatric surgery in a period of 15 years between 2003 and 2018. The inclusion criteria for this study were as follows: every patient whose medical record contained complete data from 4 months before and after the surgery should be included in data collection and 162 patients were selected from these data. However, only 96 patients had data from 1 year after the surgery and, therefore, were used in the evolution analysis.

A standard protocol created by the nutritionist was included the following items: (a) sociodemographic data; (b) anthropometric parameters (e.g., weight, height, abdominal circumference, and BMI); (c) biochemical parameters: hemoglobin, hematocrit, total proteins, albumin, serum iron, ferritin, folic acid, calcium, vitamin B_12_, other vitamins, transaminases (AST and ALT), alkaline phosphatase, and gamma-glutamyl transferase (GGT); and (d) metabolic parameters (e.g., total cholesterol, low-density lipoprotein [LDL] and high-density lipoprotein [HDL] cholesterol, triglycerides, fasting plasma glucose, and glycated hemoglobin). These blood parameters were ordered by the surgeon or other doctors from the team. The variables collected were from the preoperative and postoperative periods. It was identified by the presence of associated morbidities (e.g., DM, systemic arterial hypertension [SAH], dyslipidemia, gastroesophageal reflux disease [GERD], and hepatic steatosis) during the preoperative period and hypovitaminosis, which were corrected (within limitations) before the surgical procedure.

The classification of obesity, according to the BMI, followed the criteria recommended by WHO and the Brazilian Association for the study of obesity and MS^1^. The nutritional status was then classified into as follows: underweight (BMI < 18.5 kg/m^2^), eutrophic (BMI between 18.5 and 24.9 kg/m^2^), overweight (BMI between 25 and 29.9 kg/m^2^), obesity class I (BMI=30-34.9 kg/m^2^), obesity class II (BMI=35-39.9 kg/m^2^), and obesity class III (BMI >40 kg/m^2^).

To recognize the presence of MS, a recommendation of the Brazilian Society of Cardiology was applied, which considers the NCEP-ATP III parameters and defines this syndrome with the presence of three or more criteria: central obesity (>102 cm for men and >88 cm for women), HDL cholesterol (<40 mg/dl for men and <50 mg/dl for women), triglycerides ≥150 mg/dl (or treatment for hypertriglyceridemia), blood pressure (BP) (SBP ≥130 mmHg and/or DBP ≥85 mmHg); or treatment for arterial hypertension and blood glucose ≥100 mg/dl (or treatment for DM)^22^.

The data were input in the Microsoft Excel 2010^®^ program. For all statistical analyses, the level of significance was determined when p<0.05. The descriptive analysis was presented in absolute and percentage frequencies. The paired t-test was used to compare the means of anthropometric, biochemical, and metabolic variables in the preoperative and postoperative periods. Pearson’s χ^2^ test was used to measure the association between MS variables during the preoperative and postoperative periods.

The design of this study followed the ethical guidelines according to Resolution 466/12 from the National Council of Health, and the research project was approved by the Research Ethics Committee (CEP) of the Centro Universitário Tiradentes-UNIT-AL (number 4.014.670).

## RESULTS

Of all 162 patients analyzed, 111 were females and 51 were males. In both sexes, the greater number of bariatric surgery was recorded between the ages 30 and 39 years, of which 41.50% were women and 39.20% were men (Table 1). The surgical technique utilized was the RYBG (gastric bypass).

**Table 1 -:** Age range of patients treated with bariatric surgery.

Age (year)	Male		Female		Total	
	N	%	N	%	N	%
18-29	15	29.4	22	19.8	37	22.8
30-39	20	39.2	46	41.5	66	40.75
40-49	10	19.6	22	19.8	32	19.75
50-59	4	7.9	16	14.4	20	12.35
≥60	2	3.9	5	4.5	7	4.35

Source: Hospital Memorial Arthur Ramos-Maceió-Al (2020).

Table 2 shows that in both men and women, more than half of the patients had SAH and dyslipidemia. It is important to highlight that 73.45% of patients had hepatic steatosis before going through surgery, whereas DM and GERD were observed in less than half of the patients.

**Table 2 -:** Prevalence of morbidities associated with obesity during the preoperative and postoperative periods of bariatric surgery.

Associated morbidities	Male		Female		Total	
	Preoperative period		Postoperative period			
	N	%	N	%	N	%
Arterial hypertension						
Yes	29	56.86	58	52.25	87	53.7
No	22	43.14	53	47.75	75	46.3
Diabetes mellitus						
Yes	17	33.33	24	21.6	41	25.3
No	34	66.66	87	78.4	121	74.7
Hepatic steatosis						
Yes	41	80.4	78	70.3	119	73.45
No	10	19.6	33	29.7	43	26.55
Dyslipidemia						
Yes	33	64.7	70	63	103	63.6
No	18	35.3	41	37	59	36.4
GERD						
Yes	18	35.3	38	34.25	56	34.55
No	33	64.7	73	65.75	106	65.45

Source: Hospital Memorial Arthur Ramos-Maceió-Al (2020).

A reduction in MS was noticed in 37% of patients in both sexes only for 4 months after the surgery (Table 3).

**Table 3 -:** Comparison between the prevalence of metabolic syndrome during the preoperative period and 4 months after bariatric surgery.

Metabolic syndrome	Male					Female				
	Preoperative period		Postoperative period		p*	Preoperative period		Postoperative period		p*
	N	%	N	%		N	%	N	%	
Yes	33	64.7	9	17.65	0.000	56	50.45	19	17.1	0.000
No	18	35.3	42	82.35	0.000	55	49.55	92	82.9	0.000

Source: Hospital Memorial Arthur Ramos-Maceió-Al (2020). *Pearson’s χ^2^ test.

Table 4 presents the information related to obesity class. It was observed that before surgery, obesity class III represented 64.20% of the total patients, of which 86.25% were men and 54.50% were women. Among men, 4 months after the surgery, 47% moved to obesity class I and progressed with a reduction in BMI. As a result, 68.95% reached the status of overweight in 1 year and 10.35% reached the status of eutrophic. Among women, 4 months into the postoperative period, 43.3% moved to obesity class I; after 1 year, 52.25% progressed to overweight, and 31.35% reached the eutrophic category.

**Table 4 -:** Comparison of nutritional status during the preoperative and postoperative periods of bariatric surgery in men and women.

Male	Preoperative period		Postoperative after 4 months		Postoperative after 1 year	
	N	%	N	%	N	%
Eutrophic	0	0	0	0	3	10.35
Overweight	0	0	17	33.4	20	68.95
Obesity class I	0	0	24	47	6	20.7
Obesity class II	7	13.75	7	13.7	0	0
Obesity class III	44	86.25	3	5.9	0	0
Female	Preoperative period		Postoperative after 4 months		Postoperative after 1 year	
	N	%	N	%	N	%
Eutrophic	0	0	0	0	21	31.35
Overweight	0	0	36	32.4	35	52.25
Obesity class I	2	1.8	48	43.3	9	13.4
Obesity class II	49	44.15	22	19.8	2	3
Obesity class III	60	54.5	5	4.5	0	0

Source: Hospital Memorial Arthur Ramos-Maceió-Al (2020).

The comparison between the mean results of total weight and BMI of patients, during the preoperative and postoperative periods, revealed a reduction with a significant value in both sexes, and it continued to reduce for up to 1 year (Tables 5 and 6).

**Table 5 -:** Comparison of anthropometric, metabolic, and biochemical parameters of people with obesity during the preoperative period and 4 months after bariatric surgery.

	Male			Female		
	Preoperative period	Postoperative period	p*	Preoperative period	Postoperative period	p*
	Mean±SD	Mean±SD		Mean±SD	Mean±SD	
Anthropometric parameters						
Weight (kg)	133.87±22.25	97.36±16.72	0.000	107.5±13.41	84.39±11.50	0.000
BMI (kg/m²)	44.57±5.12	32.53±4.46	0.000	41.59±4.70	35.57±4.35	0.000
Metabolic parameters						
HDL (mg/dl)	41.98±10.12	39.18±10.26	0.064	49.67±10.79	44.88±9.45	0.000
LDL (mg/dl)	114.49±29.55	94.58±33.37	0.001	125.28±30.14	99.37±25.80	0.000
TC (mg/dl)	188.85±31.65	154.30±45.92	0.000	203.17±40.88	162.53±30.42	0.000
TG (mg/dl)	184.08±86.18	99.51±40.28	0.000	149.65±80.02	104.40±43.50	0.000
Fasting plasma Glucose (mg/dl)	107.89±35.48	81.63±8.78	0.000	95.10±27.83	84.06±24.86	0.000
Glycated Hemoglobin (%)	8.07±10.15	6.63±7.52	0.568	6.09±1.18	5.47±0.90	0.000
Biochemical parameters						
Hemoglobin (g/dl)	15±1.34	14.26±1.07	0.000	12.87±0.96	12.52±0.95	0.004
Hematocrit (%)	44.44±5.59	42.91±3.24	0.053	39.03±3.76	38.12±2.77	0.024
Total proteins (g/dl)	7.25±0.72	8.82±11.53	0.013	7.12±0.54	6.76±0.38	0.000
Albumin (g/dl)	4.28±0.45	12.37±52.34	0.325	3.99±0.41	4.01±0.38	0.406
Serum iron (μg/dl)	99.65±33.22	87.10±30.80	0.448	80.8±34.19	70.13±28.02	0.020
Ferritin (ng/ml)	351.33±286.97	251.11±136.82	0.010	102.50±247.62	90.06±90.61	0.068
Vitamin B12 (pg/ml)	395.43±126.31	394.55±152.35	0.686	365.72±151.63	383.22±159.53	0.233
Folic acid (ng/ml)	11.30±5.84	15.09±8.91	0.028	10.62±5.16	17.46±5.60	0.000
AST (U/l)	34.47±23.04	23.63±6.02	0.002	25.96±15.86	30.12±20.56	0.017
ALT (U/l)	49.75±23.92	33.01 ±16.70	0.000	31.46±32.01	33.62±26.44	0.048
Alkaline phosphatase (U/l)	68.16±23.48	49.96±25.19	0.920	76.01±29.46	118±66.92	0.172
Gamma-glutamyl transferase (U/l)	55.22±26.81	24.15±12.63	0.000	39.68±34.25	25.72±27.18	0.000
Calcium (mg/dl)	11.07±11.26	9.52±0.59	0.362	10.10±8.42	9.89±6.58	0.853

Source: Hospital Memorial Arthur Ramos-Maceió-Al (2020).

**Table 6 -:** Comparison of anthropometric, metabolic, and biochemical parameters of people with obesity in the preoperative period and 1 year after bariatric surgery.

	Male			Female		
	Preoperative period	Postoperative period	p*	Preoperative period	Postoperative period	p*
	Mean±SD	Mean±SD		Mean±SD	Mean±SD	
Anthropometric parameters						
Weight (kg)	133.87±22.25	81.11±9.60	0.000	107.75±13.41	70.71±9.76	0.000
BMI (kg/m²)	44.57±5.12	26.98±5.64	0.000	41.59±4.70	27.29±3.52	0.000
Metabolic parameters						
HDL (mg/dl)	41.98±10.12	51.66± 12.25	0.324	49.67±10.79	53.55±12.09	0.028
LDL (mg/dl)	114.49±29.55	80.99±22.71	0.001	125.28±30.14	99.11±79.43	0.023
TC (mg/dl)	188.85±31.65	149.20±25.78	0.001	203.17±40.88	155.04±34.32	0.000
TG (mg/dl)	184.08±86.18	76.36±24.00	0.000	149.65±80.02	87.35±13.08	0.000
Fasting plasma Glucose (mg/dl)	107.89±35.48	80.20±8.25	0.007	95.10±27.83	84.03±8.66	0.001
Glycated hemoglobin (%)	8.07±10.15	4.77±0.56	0.009	6.09±1.18	5.30±0.54	0.001
Biochemical parameters						
Hemoglobin (g/dl)	15±1.34	13.75±1.28	0.031	12.87±0.96	12.01±1.00	0.005
Hematocrit (%)	44.44±5.59	41.29±3.71	0.022	39.03±3.76	36.78±2.95	0.001
Total proteins (g/dl)	7.25±0.72	6.85±0.51	0.046	7.12±0.54	6.4±0.82	0.015
Albumin (g/dl)	4.28±0.45	4.22±0.28	0.298	3.99±0.41	4.04±0.34	0.394
Serum iron (μg/dl)	99.65±33.22	87.09±21.37	0.059	80. 8±34.19	83.55±32.47	0.484
Ferritin (ng/ml)	351.33±286.97	208.37±112.18	0.092	102. 50±247. 62	101.52±105.98	0.351
Vitamin B12 (pg/ml)	395.43±126.31	472.75±248.09	0.667	365. 72±151.63	293.40±113.83	0.028
Folic acid (ng/ml)	11.30±5.84	17.32±6.09	0.002	10.62±5.16	26.29±36.89	0.037
AST (U/l)	34.47±23.04	29.80±13.68	0.499	25.96±15.86	27.37±13.08	0.586
ALT (U/l)	49.75±23.92	32.62±17.29	0.060	31.46±32.01	31.46±32.02	0.631
Gamma-glutamyl transferase (U/l)	55.22±26.81	27.28±12.79	0.000	39.68±34.25	19.83±10.26	0.001
Calcium (mg/dl)	11.07±11.26	8.89±2.27	0.868	10.10±8.42	12.00±12.71	0.214

Source: Hospital Memorial Arthur Ramos-Maceió-Al (2020).

Regarding the evaluation of metabolic parameters, there was a reduction in HDL levels in both sexes for 4 months after the surgery (Table 5); however, an elevation at the end of the first year was observed (Table 6). LDL, total cholesterol, triglycerides, and fasting plasma glucose showed a significant reduction in both sexes after 4 months (Table 5). Regarding the glycated hemoglobin values, it was observed a reduction in the fourth month (Table 5), which was more relevant after 1 year (Table 6).

The biochemical parameters (e.g., AST, ALT, and GGT) presented a significant reduction in both sexes for 4 months after the intervention. Total proteins exhibited a small increase in men in the first 4 months and an insignificant reduction after 1 year. Among women, there was less reduction in both periods; however, these parameters remained in the normality range for both sexes. Hematocrit and hemoglobin did not show relevant reductions in their values after 4 months, whereas ferritin demonstrated a greater reduction. Regarding vitamin B_12_, there was almost no reduction in the fourth month; however, after 1 year, it was observed a reduction only in women. Meanwhile, folic acid demonstrated an increase during all the periods analyzed for both sexes (Tables 5 and 6).

## DISCUSSION

The Brazilian Institute of Geography and Statistics (IBGE) states that, in the country, obesity was more than doubled in the population aged 20 years or above in 2019^14^. According to the National Health Survey, obesity among women in the same age group grew from 14.5% to 30.2% and stayed higher than male statistics, which grew from 9.6% to 22.8%. The majority of patients treated with bariatric surgery in this study were female (68.50%), in agreement with findings from several national and international studies, and with greater prevalence in the age group between 30 and 39 years for both sexes ^6^ ^,^ ^21^ ^,^ ^26^.

A meta-analysis of 89 studies performed in other countries about the impact of gastroplasty in the obesity treatment showed that the mean age of patients was 38 years and that more than one-third of them were female^26^. Another meta-analysis, which involved 134 studies and 22,094 patients, demonstrated that 73% of them were female with an average age of 39 years^6^.

In addition to epidemiological data, which proved a growth and a prevalence of obesity in women, some studies also suggested that other factors might explain the predominance of bariatric surgery in women, such as personal aesthetic motivations and stigma of the society that worships beauty ideals of thin women. Different from women, men tend to search this resource only when there is a compromise in their health and daily physical activities^8^.

When the impact of weight loss after bariatric surgery was evaluated, it was noted in this study, as well as in many others, that this procedure has been shown to be highly effective^3^ ^,^ ^11^ ^,^ ^12^.

Regarding the anthropometric data, the results here presented showed a mean reduction of 36.51 kg in body weight and 12.04 kg/m^2^ in BMI in men, during the period of 4 months after gastroplasty. Meanwhile, for women, there was a less significant reduction, with a mean loss of 23.36 kg in body weight and 6.02 kg/m^2^ in BMI during the same period. One year after the surgery, there was mean reduction of 52.76 kg in body weight and 17.59 kg/m^2^ in BMI in men, whereas in women, there was a reduction of 37.04 kg in body weight and 14.3 kg/m^2^ in BMI. In addition, the eutrophic category was reached by 31.35% of women and 10.35% of men and more than half of patients reached the overweight status for both sexes.

These data implied an expressive reduction in cardiovascular mortality and in other diseases associated with excess body weight. We highlighted that this result corroborates other scientific studies, which identified positive results that significantly reduced BMI only 6 months after gastroplasty^23^ ^,^ ^24^. Meanwhile, other studies only identified such results at the end of the first year^17^ ^,^ ^20^.

This study showed that 104 (93.69%) of 111 female patients and 48 (94.11%) of 51 male patients had some kind of morbidity, indicating that obesity is a clinical condition which predisposes the development of other diseases. The most prevalent morbidities in men and women, respectively, were hepatic steatosis (80.4% and 70.3%), dyslipidemias (64.7% and 63%), MS (64.7% and 54.45%), arterial hypertension (56.86%), GERD (35.3% and 34.25%), and, DM (33.33% and 21.6%). These results were also found in other studies^3^ ^,^ ^8^.

It is known that the duodenal and jejunal “bypass” performed in the procedure progressively reduces fasting plasma glucose and improves insulin resistance. This intervention has now been called metabolic surgery based on evidence that several studies have presented with regard to its efficiency for treating DM and a significant reduction in MS for both sexes at the end of the first year after the procedure-which was also evidenced in this research^2^.

Recent studies have questioned the mechanism by which patients with morbid obesity control their DM2 after bariatric surgery. In fact, weight loss is partially responsible for reversing DM2. The intestinal derivation, which is performed in these surgeries, causes an immediate improvement effect and an increase in insulin production, due to the accentuated production of glucagon-like peptide-1 (GLP-1) and the improved action of gastric inhibitory polypeptide (GIP), or glucose-dependent insulinotropic polypeptide, which contribute to the improvement of plasma glucose levels and, consequently, of DM2^25^.

Therefore, the relevant reduction in body weight and visceral fat through gastroplasty determines, in short term, a positive impact on the control/resolution of comorbidities associated with obesity, including MS, DM, dyslipidemia, SAH, hepatic steatosis, and cardiovascular diseases, and, as consequence, increases life expectancy. Thus, bariatric surgery, currently, is the choice of treatment for severe obese people in which behavioral-pharmacological therapy was not effective^15^.

However, malnutrition and nutritional deficiency are common after this procedure. In RYGB, there is a higher prevalence of vitamin B_12_, iron, and folic acid deficiencies. These deficiencies generally occur because of feeding restrictions, physiological impacts of anatomic changes, as well as food intolerances and nonadherence to therapy with multivitamins^5^.

Considering these facts, in this study, patients treated with bariatric surgery were regularly followed up by a physician and a nutritionist in order to detect possible nutritional deficiencies and were individually given vitamin and mineral supplements since the preoperative period. Consequently, it was not observed an expressive reduction in these nutrients. Vitamin B_12_ deficiency can occur as a consequence of several factors, such as the reduction of gastric production of hydrochloric acid, which does not promote the conversion of pepsinogen in pepsin, that is necessary for the release of vitamin B_12_ in protein-rich foods. In addition, intrinsic factor is produced by parietal cells in the stomach. When there is no production of intrinsic factor, or this production is insufficient as it happens with the reduction of the gastric compartment, there is no absorption of vitamin B_12_ at the terminal ileum, which then originates pernicious anemia^5^.

Low levels of vitamin B_12_ can be observed 6 months after surgery; however, in the majority of times, this occurs after 1 year or more, once its liver storage is depleted-which was evidenced in our study for female patients^5^.

Dyslipidemia was present in the majority of patients for both sexes in this study and showed a significant reduction in TC, LDL, and TG during the first year after the surgery. Meanwhile, HDL demonstrated an increase in its levels for only 4 months after the surgery.

Finally, studies have unquestionably demonstrated the efficacy of gastroplasty in the treatment of obesity^5^ ^,^ ^23^. It is known that triglyceride levels are consistently reduced immediately after the surgery and their levels are maintained in the long term, even if the patient regains weight^20^. Reductions of higher magnitude are observed after gastric bypass surgery, which is both restrictive and absorption limiting^24^. Therefore, the reduction is a result of the change in food habits that are characterized by the ingestion of small food quantities and with less calories, which promotes the adoption of healthier life habits ^21^.

## CONCLUSION

The results corroborate the importance of persisting in nutritional and behavioral actions for these patients, which will contribute to the achievement and maintenance of an ideal weight, while avoiding weight regain that may force them to return to risk situations, that is, before the gastroplasty.

The positive impact resulted from gastroplasty on weight loss, BMI reduction, and improvement of metabolic and biochemical parameters is already extremely significant 4 months after the surgery, and it is maintained until the end of the first year after the surgical procedure, even in patients who did not fit the eutrophic category.
